# Assessing the utility of the Oxford Nanopore MinION for snake venom gland cDNA sequencing

**DOI:** 10.7717/peerj.1441

**Published:** 2015-11-24

**Authors:** Adam D. Hargreaves, John F. Mulley

**Affiliations:** 1 Department of Zoology, University of Oxford, Oxford, United Kingdom; 2 School of Biological Sciences, Bangor University, Bangor, United Kingdom

**Keywords:** Nanopore, MinION, Venom gland, cDNA, Viper, Echis, Transcriptomics, Venom, Sequencing

## Abstract

Portable DNA sequencers such as the Oxford Nanopore MinION device have the potential to be truly disruptive technologies, facilitating new approaches and analyses and, in some cases, taking sequencing out of the lab and into the field. However, the capabilities of these technologies are still being revealed. Here we show that single-molecule cDNA sequencing using the MinION accurately characterises venom toxin-encoding genes in the painted saw-scaled viper, *Echis coloratus*. We find the raw sequencing error rate to be around 12%, improved to 0–2% with hybrid error correction and 3% with *de novo* error correction. Our corrected data provides full coding sequences and 5′ and 3′ UTRs for 29 of 33 candidate venom toxins detected, far superior to Illumina data (13/40 complete) and Sanger-based ESTs (15/29). We suggest that, should the current pace of improvement continue, the MinION will become the default approach for cDNA sequencing in a variety of species.

## Introduction

The transcriptome can be defined as all of the RNA molecules expressed by a cell or population of cells, for example in a particular tissue (McGettigan, 2013). As this includes all expressed mRNA molecules, the transcriptome can be inferred to represent all protein coding genes that are actively transcribed at the time of sampling (Rudd, 2003). In theory then, the transcriptome is the precursor to the proteome of a cell or tissue, although post-transcriptional and post-translational modification and regulation are likely to cause some disparity between the two. Traditionally transcriptomes were analysed via cloning and sequencing of expressed sequence tags (ESTs) whereby short fragments of a cDNA library are sequenced and clustered to give a contiguous sequence. ESTs are ultimately limited by their short length (typically 200–800 bp) (Nagaraj, Gasser & Ranganathan, 2007) and low coverage, meaning lowly expressed transcripts and splice variants are likely to remain undetected (Rudd, 2003). The advent of “next-generation” sequencing technologies such as the Roche 454, ABI SOLiD and Illumina Genome Analyzer platforms in the first decade of the 21st century facilitated a step-change in transcriptome studies: increased sequencing depth improves the likelihood of recovering full-length transcript sequences (including lowly expressed transcripts), and higher resolution aids in the identification of splice variants. As the number of reads sequenced from a particular transcript will be representative of the amount of that transcript present in a sample, such data is also quantitative (Marguerat & Bahler, 2010). Both the ABI SOLiD and Roche 454 systems are no longer available/supported, and the DNA sequencing market is now largely dominated by platforms that produce high numbers of short reads. The assembly of these reads into full transcript sequences poses several challenges, especially in the absence of a reference genome. Unlike the genome (which remains relatively static), the transcriptome can be highly variable, with mRNA transcripts encoding different genes present at different abundances within a given sample, resulting in uneven sequencing coverage (Rudd, 2003; Sims et al., 2014), particularly in highly transcriptionally active tissues. The short read length also means that reads from highly similar transcripts, such as paralogs (members of a gene family produced by gene duplication, as distinct from orthologs which are produced via speciation) belonging to the same gene family, may be fused during the assembly process resulting in chimeric sequences. Alternative transcripts of the same gene may be omitted altogether if the abundance of one variant in a sample significantly outweighs the other(s) (Martin & Wang, 2011) and, finally, shared homologous sequences in related genes may be incorporated or omitted erroneously, especially if they are highly conserved.

The characterisation of the venom gland transcriptomes of venomous snakes has been particularly useful in revealing the genetic basis of inter- and intra-specific variation in venom composition, something which has significant implications for antivenom manufacture (Fry et al., 2001; Casewell et al., 2014; Sunagar et al., 2014; Gutierrez et al., 2010). Although genome sequences for some venomous species are now available (including the king cobra *Ophiophagus hannah* (Vonk et al., 2013) and the speckled rattlesnake, *Crotalus mitchellii* (Gilbert et al., 2014)), for the vast majority of species *de novo* assembly of short-read sequences has been the only feasible (and cost-effective) approach. However, such approaches have difficulty in accurately reconstructing full-length sequences for highly similar paralogs in some key venom gene families. For example, we have previously found that assemblies of Illumina HiSeq data using Trinity (version trinityrnaseq_r2012-04-27, (Grabherr et al., 2011)) only provided full-length coding sequences for 13 candidate venom toxin encoding genes in the painted saw-scaled viper (*Echis coloratus*) (Hargreaves et al., 2014b; Hargreaves et al., 2014a). Others have shown similar issues with venom gland transcriptomes from the Okinawa habu (*Protobothrops flavoviridis*) and the Hime habu (*Ovophis okinavensis*), where 37/103 and 29/95 complete transcripts were identified respectively (Aird et al., 2013). Attempts have been made to develop an assembler specifically for samples containing large numbers of highly similar transcripts, such as VTbuilder (Archer et al., 2014), although the current version has an upper limit of 5 million ≥ 120 bp reads, making it less suitable for the analysis of large-scale data generated from the most recent Illumina platforms or for the re-analysis of older datasets with shorter read lengths. Long-read data derived from single-molecule sequencing should eliminate many of the current problems associated with the investigation of snake venom gland transcriptomes, but the only currently commercially-available long-read platform (the Pacific Biosciences RSII) typically requires a large number of flowcells (12–16 for a comprehensive survey of full-length isoforms, each costing £400) and several size-selection and PCR steps.

**Figure 1:**
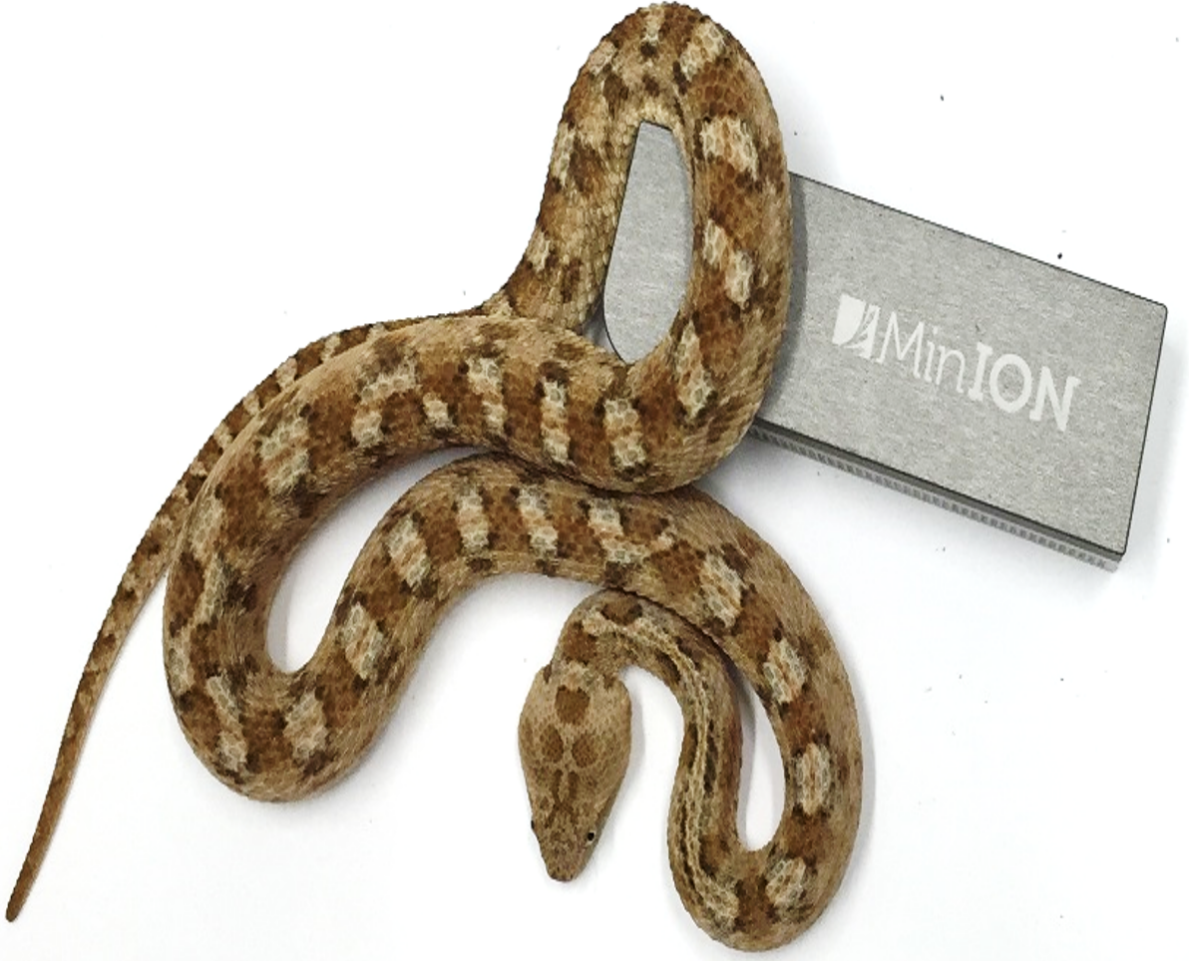
The Oxford Nanopore MinION portable DNA sequencing device and a painted saw-scaled viper, *Echis coloratus*.

The Oxford Nanopore MinION (Fig. 1) is a portable, USB 3.0-powered DNA sensing device that uses an application-specific integrated circuit (ASIC) to detect miniscule voltage changes resulting from the movement of DNA strands through pores embedded in a membrane. The disposable flowcell (£300–500 each depending on quantity purchased) contains 2,048 sensor wells (each of which contains a single pore), with 512 measurement channels below these. The choice of which is the “best” pore to use is performed by the multiplexer (or “mux”) during an initial platform QC step, and the standard 48 h run protocol performs one switch to an alternative pore after 24 h. A “motor” protein unwinds the DNA as it enters the pore and controls the speed at which the DNA translocates the pore to facilitate accurate base-calling and a “hairpin” adaptor at the other end of the DNA enables both strands to be read. Since the same piece of DNA is analysed twice, a consensus (“2D”) read of greater accuracy can therefore be generated. The MinION was initially made available to selected users in the MinION Access Program (MAP) in spring 2014, with the first publications emerging in late 2014/early 2015 and the rapid dissemination of results and protocols facilitated by an active online community and preprint servers such as bioRxiv (http://biorxiv.org). The utility of the MinION for the rapid and accurate investigation of disease outbreaks (Quick et al., 2015; Check Hayden, 2015); microbial diversity analysis (Kilianski et al., 2015); sequencing of bacterial and viral genomes (Quick, Quinlan & Loman, 2014; Madoui et al., 2015; Wang et al., 2015; Kilianski et al., 2015), haplotype resolution (Ammar et al., 2015) and even for the characterisation of more complex eukaryotic genomes (Goodwin et al., 2015) has already been demonstrated. However, the utility of this device for the characterisation of transcriptomes has not yet been comprehensively investigated (a previous study investigating the venom gland transcriptome of the Okinawa habu (*Protobothrops flavoviridis*) was based on an amplicon sequencing protocol, and produced very small amounts of data from a single flow cell, (Table 1) (Mikheyev & Tin, 2014)). We therefore set out to establish the feasibility of using the Oxford Nanopore MinION to characterise snake venom gland transcriptomes, something for which long-read data derived from single DNA molecules should be eminently suitable, and which should help to overcome the issues associated with *de novo* assembly of highly similar venom gene paralogs. We chose to investigate the painted saw-scaled viper, *Echis coloratus* (Fig. 1), as this species is not only a member of the genus of snakes thought to be responsible for more deaths than any other (Casewell et al., 2009; Warrell et al., 1977), but it is also one for which we have Illumina HiSeq data (Hargreaves et al., 2014a; Hargreaves et al., 2014b) and for which ESTs derived from Sanger (dideoxy, chain-termination) sequencing are available (Casewell et al., 2009).

**Table 1:** Oxford Nanopore MinION venom gland transcriptome sequencing statistics. Painted saw-scaled viper (*Echis coloratus*) data was derived from two individuals (Eco6 and Eco8), using four R7.3 flowcells and both the standard 48 h run (with a “re-mux” voltage change at 24 h) and a modified run utilising four re-mux steps at 8 h intervals. *Protobothrops flavoviridis* statistics are derived from a reanalysis of the raw data of Mikheyev & Tin (2014). ‘Pass’ data is that selected by the base-calling software Metrichor as being high quality and consists entirely of 2D read data.

		Eco6 (48 h)	Eco8 (48 h)	Eco6 (4 × 8 h)	Eco8 (4 × 8 h)	*Protobothrops flavoviridis*
	Available pores	436	332	345	387	(Unknown)
All data	Total reads	93,697	47,068	66,916	58,628	2,057
	Total bases (Mb)	132.1	70.3	81.7	80.1	1.3
	Max length (bp)	454,436	278,051	363,606	212,026	29,363
	Min length (bp)	5	5	5	7	5
	Mean length (bp)	1,410	1,493	1,220	1,378	614
	N50 (bp)	1,577	1,753	1,412	1,648	823
‘Pass’ data only	Total reads	16,804	7,190	9,172	7,786	16
	Total bases (Mb)	22.7	11	12.2	11.7	0.019
	Max length (bp)	12,639	5,869	10,422	8,521	2,195
	Min length (bp)	247	251	287	248	650
	Mean length (bp)	1,352	1,536	1,333	1,509	1,220
	N50 (bp)	1,536	1,801	1,504	1,782	1,323

## Methods

### mRNA extraction and double-stranded cDNA synthesis

Total RNA was extracted from the venom glands of two *Echis coloratus* (snap-frozen after removal and stored at −80 °C (Hargreaves et al., 2014a; Hargreaves et al., 2014b)) using TriReagent (Sigma T9424; Sigma Aldrich, St. Louis, MO, USA) and mRNA purified using the polyA Spin mRNA Isolation Kit (New England BioLabs S1560; New England BioLabs, Ipswich, MA, USA). mRNA was quantified using a Qubit fluorometer (Qubit RNA HS Assay Kit Q32852; Thermo Scientific, Waltham, MA, USA) and reverse transcription carried out using 120 ng (Eco6) or 240 ng of mRNA (Eco8). Primer annealing was performed at 65 °C for 5 min in a 13 µl reaction comprising the required amount of mRNA, 2 µl of 1 µM Oligo d(T)23 VN primer (New England BioLabs S1327S; New England BioLabs, Ipswich, MA, USA), 1 µl of 10 mM dNTPs and the appropriate volume of Rnase-free water. The reaction was then snap-cooled on a pre-chilled freezer block. 4 µl of 5× First Strand buffer and 2 µl 100 mM DTT (part of Life Technologies 18064-014; Life Technologies, Carlsbad, CA, USA) were then added to the primer/mRNA mix, which was briefly vortexed, spun down in a microcentrifuge and incubated at 42 °C for 2 min. Finally, 1 µl of 200 U/µl SuperScript II Reverse Transcriptase (Life Technologies 18064-014; Life Technologies, Carlsbad, CA, USA) was added to each tube and reverse transcription carried out at 50 °C for 50 min, with a subsequent 15 min incubation at 70 °C for enzyme denaturation. Second strand synthesis was performed with the NEBNext mRNA Second Strand Synthesis Module (New England BioLabs E6111; New England BioLabs, Ipswich, MA, USA), using 45 µl of nuclease-free water, 10 µl of NEBNext Second Strand Synthesis Reaction Buffer and 5 µl of NEBNext Second Strand Synthesis Enzyme Mix, with incubation at 16 °C for 1 h. Double-stranded cDNA (ds cDNA) was purified using a 1.8× volume of Agencourt AMPure XP beads (Beckman Coulter A63880), with a 5 min binding step (with gentle shaking), two washes in 200 µl 70% ethanol and elution in 51 µl nuclease-free water.

### End-repair and dA-tailing

End-repair was performed using the NEBNext End Repair Module (New England BioLabs E6050; New England BioLabs, Ipswich, MA, USA) with 6 µl of 10× end-repair buffer and 3 µl of end-repair enzyme mix added to each of the 51 µl ds cDNA samples, followed by incubation at room temperature for 25 min and clean-up using a 1.8× volume of Agencourt beads (as above), with elution in 25 µl nuclease-free water. Next, the end-repaired ds cDNA was dA-tailed with the NEBNext dA-Tailing Module (New England BioLabs E6053; New England BioLabs, Ipswich, MA, USA), using 3 µl of 10× NEBNext dA-Tailing Reaction Buffer and 2 µl of A-tailing enzyme (Klenow Fragment (3′ → 5′ exo-)) and incubation at 37 °C for 30 min, followed by clean-up with 1.8× Agencourt beads (as above) and elution in 15 µl of nuclease-free water.

### PCR adapter ligation and amplification

Prior to amplification, adapters were ligated to the end-repaired, dA-tailed ds cDNA using 5 µl of the Oxford Nanopore SQK-MAP005 PCR adapters (a double-stranded oligonucleotide supplied by Oxford Nanopore, formed by heating a solution containing each oligo (Short_Y_top_LI32 5′-GGTTGTTTCTGTTGGTGCTGATATTGCGGCGTCTGCTTGGGTGTTTAACCT-3′ and Y_bottom_LI33 5′-GGTTAAACACCCAAGCAGACGCCGAAGATAGAGCGACAGGCAAGTTTTGAGGCGAGCGGTCAA-3′) at 20 µM in 50 mM NaCl, 10 mM Tris–HCl pH7.5 to 95 °C for 2 min, and cooling by 0.1 °C every 5 s) and 20 µl of Blunt/TA Ligase Master Mix (New England BioLabs M0367; New England BioLabs, Ipswich, MA, USA), with incubation at room temperature for 15 min. Adapter-ligated DNA was purified using 0.7× of Agencourt beads (as above) and eluted in 25 µl nuclease-free water, followed by amplification using 50 µl of LongAmp Taq 2× master mix (New England BioLabs M0287; New England BioLabs, Ipswich, MA, USA), 2 µl of Oxford Nanopore SQK-MAP005 PCR primers (PR2 5′-TTTCTGTTGGTGCTGATATTGC-3′ and 3580F 5′-ACTTGCCTGTCGCTCTATCTTC-3′) and 23 µl nuclease-free water. Initial denaturation was 95 °C for 3 min, followed by 15 cycles of 95 °C for 15 s, 62 °C for 15 s and 65 °C for 5 min, with a final extension at 65 °C for 10 min. Amplified DNA was purified using 0.7× Agencourt beads (as above) with elution in 80 µl of nuclease-free water.

### Sequencing adapter ligation

End-repair of the amplified DNA was carried out using the NEBNext End Repair Module (New England BioLabs E6050; New England BioLabs, Ipswich, MA, USA), with 10 µl of 10× end-repair buffer, 5 µl of end-repair enzyme mix and 5 µl of nuclease-free water and incubation at room temperature for 20 min. End-repaired DNA was purified using 1× volume of Agencourt beads as outlined previously, with elution in 25 µl of nuclease-free water. dA-tailing and clean-up was carried out as described above, with elution in 30 µl of nuclease-free water. Adapter ligation was performed for 10 min at room temperature in Protein LoBind 1.5 ml Eppendorf tubes (Sigma Aldrich Z666505-100EA; Sigma Aldrich, St. Louis, MO, USA) using 10 µl of each of the Oxford Nanopore SQK-MAP005 adapter and HP adapters and 50 µl of Blunt/TA Ligase Master Mix (New England BioLabs M0367). Clean-up was performed using an equal volume of Dynabeads His-Tag Isolation and Pulldown beads (Life Technologies 10103D; Life Technologies, Carlsbad, CA, USA), which had been washed twice in SQK-MAP005 1× Bead Binding Buffer and resuspended in 100 µl of 2× Bead Binding Buffer. The bead/DNA mix was incubated at room temperature for 5 min to allow binding, washed twice in 200 µl of 1× Bead Binding Buffer, eluted in 25 µl of elution buffer and the resulting ‘Pre-sequencing library’ either used immediately or stored at −20 °C in 6 µl aliquots in LoBind tubes.

### Flowcell preparation and sample loading

A total of four Oxford Nanopore FLO-MAP003 (R7.3) flowcells were used, and these were stored at 4 °C from delivery until use. Flowcells were fitted into MIN-MAP001 MinION Sequencing Devices and secured using the provided nylon screws and new heat pads were used for each flowcell. Prior to sample loading, the flowcells were primed using two 10 min washes of 150 µl of 1× SQK-MAP005 Running Buffer with 3.25 µl of Fuel Mix. Finally, a 6 µl aliquot of the pre-sequencing library was mixed with 75 µl of 2× Running Buffer, 66 µl of nuclease-free water and 3 µl of Fuel Mix then briefly mixed by inversion, microfuged and loaded onto the flowcell.

### Sequencing

Sequencing utilised both the standard 48-hour sequencing protocol and a modified 4× 8-hour protocol (J Tyson, pers. comm., 2015), run using the MinKNOW software (version 0.49.2.9). For the 48 h runs, a fresh aliquot of sequencing library was added at around 24 h. Base-calling from read event data was performed by Metrichor (version 2.26.1) using the 2D basecalling workflow (version 1.14). We also re-analysed the Okinawa habu (*Protobothrops flavoviridis*) venom gland data of Mikheyev & Tin (2014) using this Metrichor version and workflow.

### Data analysis

Sequencing statistics were determined and data extracted in .fastq and .fasta format using poretools (Loman & Quinlan, 2014) and poRe (Watson et al., 2015). Error correction was carried out using both hybrid and *de novo* correction methods. Hybrid error correction using short-read (2 × 100 bp paired-end reads) sequencing data previously generated on the Illumina HiSeq platform was carried out using a module of proovread (Hackl et al., 2014). More specifically, we utilised proovread-flex, which is optimised for the uneven sequencing coverage seen in metagenomes and transcriptomes. For *de novo* error correction we utilised nanocorrect (Loman, Quick & Simpson, 2015) (available at https://github.com/jts/nanocorrect) using commands based on the full pipeline script found at https://github.com/jts/nanopore-paper-analysis/blob/master/full-pipeline.make. A single round of correction was carried out for each individual and multiple rounds trialled on Eco6 data only. We also used nanopolish (Loman, Quick & Simpson, 2015) which corrects based on the electrical signal events recorded in the original .fast5 file of the MinION read, using commands found at https://github.com/jts/nanopolish. Sequence accuracy was assessed using BWA-MEM (Li, 2013) alignments and python scripts found at https://github.com/arq5x/nanopore-scripts following Loman, Quick & Simpson (2015), assembly quality was determined using TransRate (Smith-Unna et al., 2015) and putative protein-coding open-reading frames predicted using TransDecoder (Haas et al., 2013). Corrected reads of interest were identified with BLAST+ (version 2.2.29 (Camacho et al., 2009)) using query sequences from a previously generated reference venom gland transcriptome assembly (Hargreaves et al., 2014a; Hargreaves et al., 2014b). Sequences were aligned using CLUSTAL (Larkin et al., 2007) and manually annotated to identify the protein coding ORF and 5′ and 3′ UTRs.

### Data access

Raw MinION venom gland data has been deposited in the European Nucleotide Archive under study number PRJEB10285 (Eco6 48 h run ERR985427; Eco6 4 × 8 h run ERR986484; Eco8 48 h run ERR985428; Eco8 4 × 8 h run ERR985429) and previously generated short-read sequencing data for Eco6 and Eco8 venom gland samples (Hargreaves et al., 2014a; Hargreaves et al., 2014b) can be obtained from the SRA database under the accessions ERS094900 and SRX543069 respectively.

## Results and Discussion

**Figure 2:**
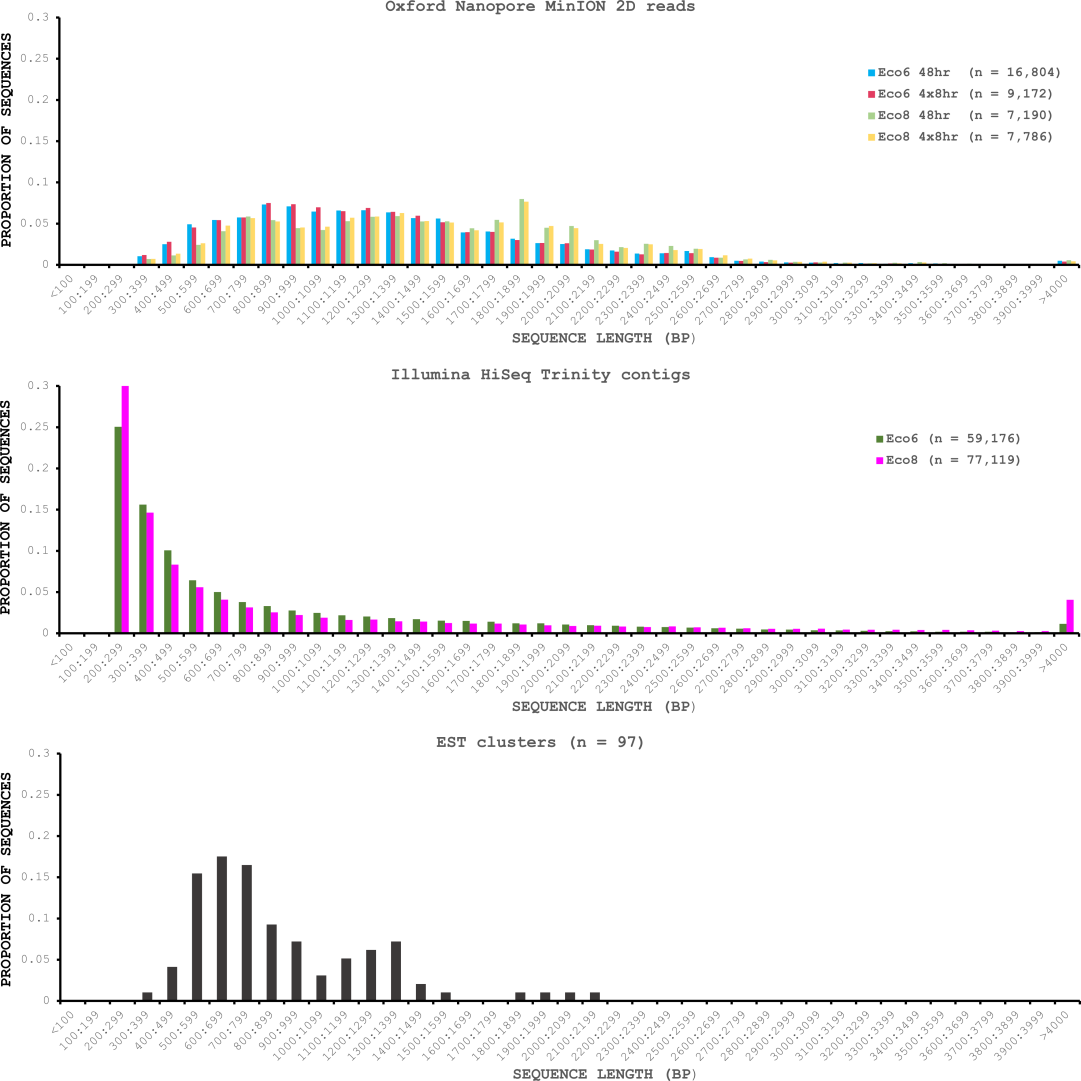
Length distributions of painted saw-scaled viper venom gland sequence data derived from multiple approaches. The Oxford Nanopore MinION data is based only on high quality reads from the Metrichor ‘pass’ folder and is derived from two individuals (Eco6 and Eco8). Both the standard 48 h sequencing protocol (which performs a re-mux after 24 h) and a modified protocol with four re-mux steps at 8 h intervals were used. Illumina HiSeq data derived from the same venom gland tissue samples was assembled using Trinity (version trinityrnaseq_r2012-04-27) and the total number of contigs is indicated for each sample. EST data are from Casewell et al. (2009), based on 1,070 Sanger reads, grouped into 97 clusters.

**Figure 3:**
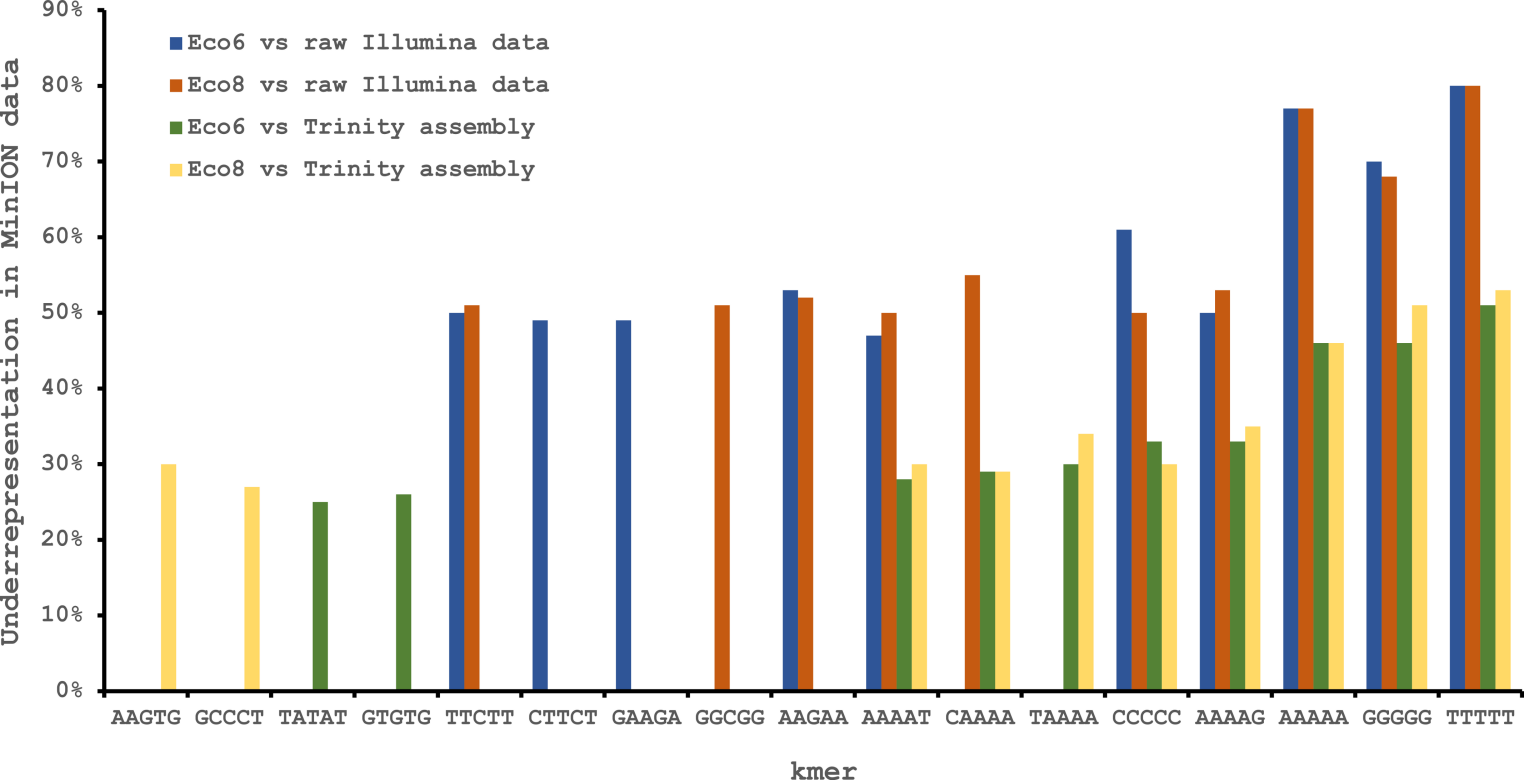
Under-represented kmers in raw Oxford Nanopore MinION data (with pooled runs for each individual) compared to raw and assembled (Trinity version trinityrnaseq_r2012-04-27) Illumina data from the same tissue samples. The ten most under-represented 5mers for each comparison are shown, with homopolymer 5mers particularly under-represented.

We used four R7.3 flowcells to characterise the venom gland transcriptome of *Echis coloratus,* using venom gland tissue samples from two individuals (“Eco6” and “Eco8”) for which we had previously generated data on the Illumina HiSeq platform (Hargreaves et al., 2014b; Hargreaves et al., 2014a). We used both the standard Oxford Nanopore 48 h run script (which performs a voltage “re-mux” after 24 h) and a set of modified scripts (J Tyson, pers. comm.) which perform four re-mux steps at 8 h intervals. Of the 512 theoretically available pores per flowcell, initial platform QC showed between 332 and 436 as actually being available for sequencing (Table 1)—figures within the range seen by many other participants of the MAP. Base-calling of data derived from the MinION is performed by cloud-based software called Metrichor and the resulting sequence data (in .fast5 format) is divided into ‘pass’ and ‘fail’ folders. The contents of the ‘fail’ folder are typically 1D and low-quality 2D data and the ‘pass’ folder contains only high-quality 2D reads. We have chosen to focus only on these high-quality ‘pass’ reads for our analyses. Our four runs generated between 7,190 and 16,804 high quality 2D reads, comprising 11–22.7 Mb of sequence, with a mean length of 1,333–1,536 bp and an N50 of 1,504–1,801 bp (Table 1). The length distribution of these reads (Fig. 2) shows a far lower proportion of short sequences than our Trinity assembly of Illumina HiSeq data derived from the same tissue samples, and also improves upon the EST cluster lengths of Casewell et al. (2009), derived from pooled venom gland samples from 10 individuals. LAST alignment (Kielbasa et al., 2011) of the ‘pass’ reads against a Trinity assembly of Illumina HiSeq data (Table 2) suggests a raw error rate in the region of 12% and the majority of errors are insertions or deletions (Table 3) (Mikheyev & Tin, 2014; Ashton et al., 2015). Based on comparisons of multiple reads from the same transcript, these errors do not appear to be systematic. Since measured current is interpreted by the basecalling software Metrichor as 5mers we also investigated the percentage change in 5mer representation between our MinION data compared to raw and assembled Illumina data for the same samples. Although crude, this analysis reveals under-representation of homopolymer 5mers (Fig. 3) (Ashton et al., 2015; Loman, Quick & Simpson, 2015). Interestingly, this pattern was not seen when we compared the MinION data to EST sequences derived from Sanger sequencing, nor was there any obvious correlation between the results obtained from Eco6 and Eco8, suggesting that the small size of this dataset (1,070 reads) is complicating these analyses.

**Table 2:** Sequence and assembly statistics for painted saw-scaled viper (*Echis coloratus*) venom gland RNA-Seq and expressed sequence tag (EST) data. Statistics are provided for two *de novo* RNA-Seq assemblers (Trinity and SOAPdenovo-trans (Xie et al., 2014)) and one genome-guided assembly method (the Tuxedo suite (Trapnell, Pachter & Salzberg, 2009)) for which we used a low coverage (∼30×) draft *E. coloratus* genome assembly. EST statistics are based on data from Casewell et al. (2009).

	Illumina HiSeq Trinity assembly		Illumina HiSeq SOAPdenovo-trans		Illumina HiSeq Tuxedo (genome-guided)		ESTs
	Eco6	Eco8	Eco6	Eco8	Eco6	Eco8	
Number of reads	13,468,544 (Eco6); 38,711,180 (Eco8)						1070
Number of bases	2,693,708,800 (Eco6); 7,819,658,360 (Eco8)						676,396
Number of contigs	59,176	77,119	136,903	169,750	33,917	48,912	97
Max length (bp)	9,014	16,826	8,331	12,403	14,002	14,007	2,162
N50 (bp)	1,619	2,338	1,175	2,034	1,625	1,683	652

**Table 3:** Correction of Oxford Nanopore MinION sequence derived from the painted saw-scaled viper (*Echis coloratus*) venom gland using proovread and nanocorrect. These approaches reduce the error rate from around 12% to 0–2% and around 4.5% (3% after a second round of correction) respectively. MinION data for the separate runs for the two *E. coloratus* individuals (Eco6 and Eco8) has been pooled.

	Uncorrected		Proovread		Nanocorrect	
	Eco6	Eco8	Eco6	Eco8	Eco6	Eco8
Total reads	25,976	14,976	21,751	11,066	7,357	4,762
Total bases (Mb)	34.9	22.7	26.1	14.6	11.4	8.1
Length (bp)						
Max	12,639	8,521	5,084	5,362	4,957	4,702
Min	247	248	300	153	19	330
N50	1,525	1,792	1,334	1,527	1,577	1,804
Alignment length (bp)	22,512,857	14,029,072	24,272,630	13,311,248	9,948,507	6,611,836
Matches	20,421,908	12,647,267	24,129,077	13,099,487	9,623,049	6,347,134
Mismatches	751,390	515,464	70,120	140,336	98,888	98,961
Insertions	663,396	416,137	9,980	12,873	75,180	48,744
Deletions	1,339,559	866,341	73,433	71,425	226,570	165,741
Total errors	2,754,345	1,797,942	153,533	224,634	400,638	313,446
	**(12.2%)**	**(12.8%)**	**(0.6%)**	**(1.7%)**	**(4.0%)**	**(4.7%)**

Hybrid error correction of our MinION reads with higher-quality short read (100 bp) Illumina data using proovread (Hackl et al., 2014) reduced the error rate to between 0 and 2%, with particular reduction in the number of indels relative to mismatches (Table 3). However, for many applications, this type of high coverage short-read data may not be available for error correction, and so we also investigated the feasibility of *de novo* error correction with nanocorrect (Loman, Quick & Simpson, 2015) using only MinION-derived reads for each individual. This approach reduced the error rate to around 4–5% using one round of correction (Table 3), and to around 3% using two rounds, with little to no further improvement seen after subsequent rounds of correction (File S1). However, the number of reads post-correction was greatly reduced and many key venom gene families of interest were missing or underrepresented. Finally, we attempted error correction using nanopolish (Loman, Quick & Simpson, 2015), a signal-level consensus algorithm which uses a hidden Markov model to correct assemblies using the original MinION electric current signals, but find that this approach performs poorly compared to both proovread and nanocorrect, giving an error rate of around 7.5%.

**Figure 4:**
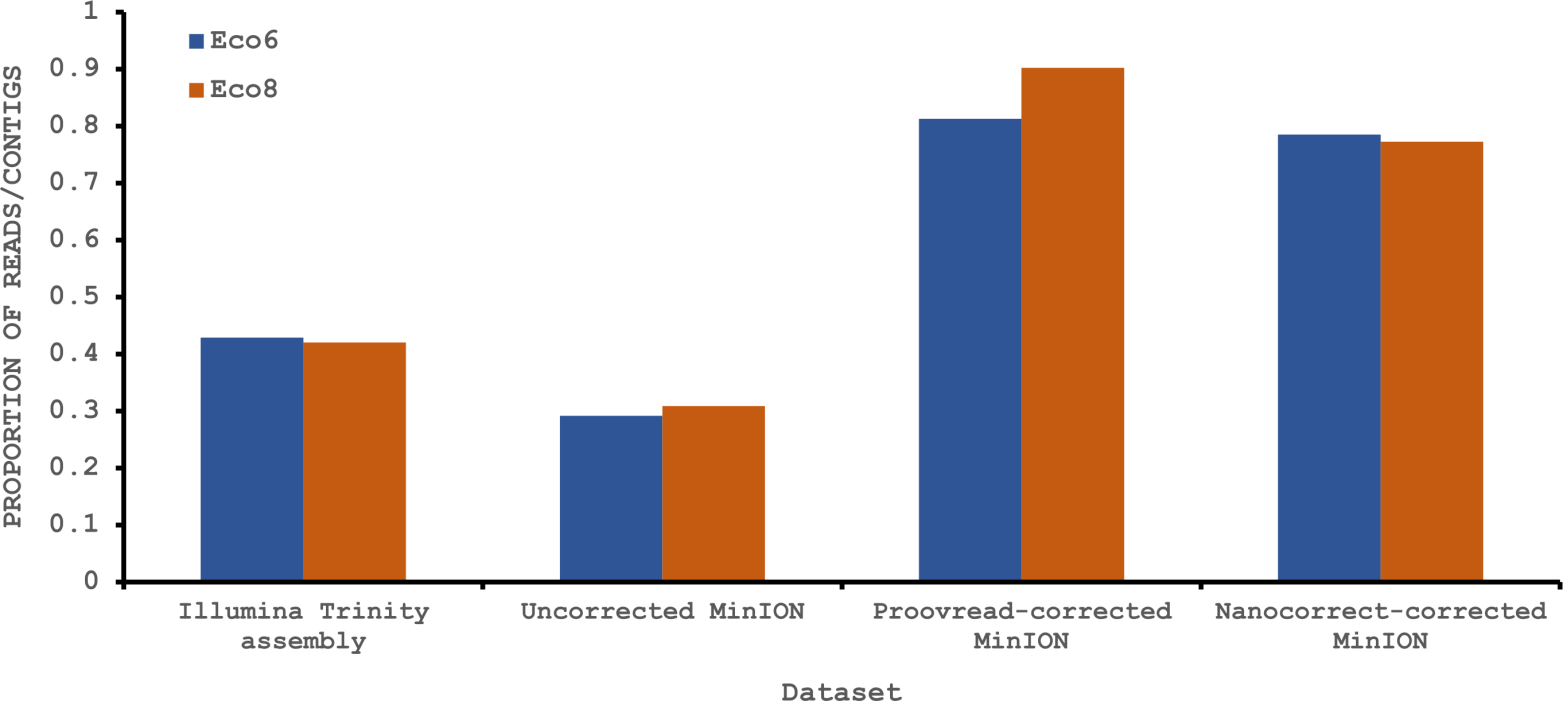
Proportion of Illumina contigs and Oxford Nanopore MinION reads with a predicted mRNA encoding an open reading frame of at least 100 amino acids that has homology to a known protein in the Swissprot protein database. Both hybrid and *de novo* correction greatly increases the proportion of MinION-derived reads with ≥100 amino acid ORF.

To provide some indication of the quality of our Illumina and corrected MinION “assemblies” we used TransRate (Smith-Unna et al., 2015), which assigns overall and optimised quality scores for *de novo* assemblies. An overall score of 0.22 and an optimised score of 0.35 have been suggested to be better than 50% of *de novo* assemblies from NCBI Transcriptome Shotgun Assembly (TSA) database (Smith-Unna et al., 2015). Our original Trinity assemblies exceed these numbers, as does the Eco8 proovread-corrected dataset (Table 4). The Eco6 proovread-corrected data has an optimised score of 0.42, but an overall score of only 0.13. Whilst it seems likely that the proovread-corrected MinION data quality is similar in quality to those derived from Illumina data, the utility of TransRate for the assessment of corrected MinION “assemblies” will require the analysis of a larger number of datasets, and we include these statistics here mainly for completeness. We next investigated putative protein coding sequences using TransDecoder (version 2.0.1) (Haas et al., 2013), specifying that any potential open reading frame (ORF) must code for a protein at least 100 amino acids long. The longest putative ORFs were compared to the Swissprot protein database (downloaded on 29/07/2015 from www.uniprot.org) and all ORFs with homology to known proteins retained (Table 4). The corrected MinION data had a higher proportion of predicted mRNAs encoding a ≥100 amino acid protein (Fig. 4) and, given the higher values for the proovread-corrected data, and the fact that it contains a greater proportion of key venom gene families, we therefore focussed on this dataset for a more detailed analysis of candidate venom toxin encoding genes in *E. coloratus*.

**Table 4:** Predicted mRNA sequences and open reading frames (ORFs) as determined by TransDecoder (Haas et al., 2013) and quality scores as determined by TransRate (Smith-Unna et al., 2015). MinION data for the separate runs for the two *E. coloratus* individuals (Eco6 and Eco8) has been pooled.

	Illumina Trinity assembly		Uncorrected nanopore		Proovread-corrected nanopore		Nanocorrect-corrected nanopore	
	Eco6	Eco8	Eco6	Eco8	Eco6	Eco8	Eco6	Eco8
Total reads/contigs	59,176	77,119	25,976	14,976	21,751	11,066	7,357	4,762
Read/contig N50 (bp)	1,492	2,142	1,525	1,792	1,334	1,527	1,577	1,804
Predicted mRNAs	25,395	32,424	7,587	4,628	17,685	9,985	5,779	3,679
mRNA N50 (bp)	2,235	3,311	1,738	1,899	1,443	1,708	1,731	1,864
Full length ORFs	7,985	14,867	5,339	3,461	6,616	4,564	4,409	2,887
ORF N50 (aa)	1,044	1,506	372	375	819	777	405	390
TransRate score	0.25	0.35	0.06	0.07	0.13	0.32	0.05	0.09
Optimal score	0.47	0.47	0.21	0.20	0.42	0.41	0.16	0.19

**Figure 5:**
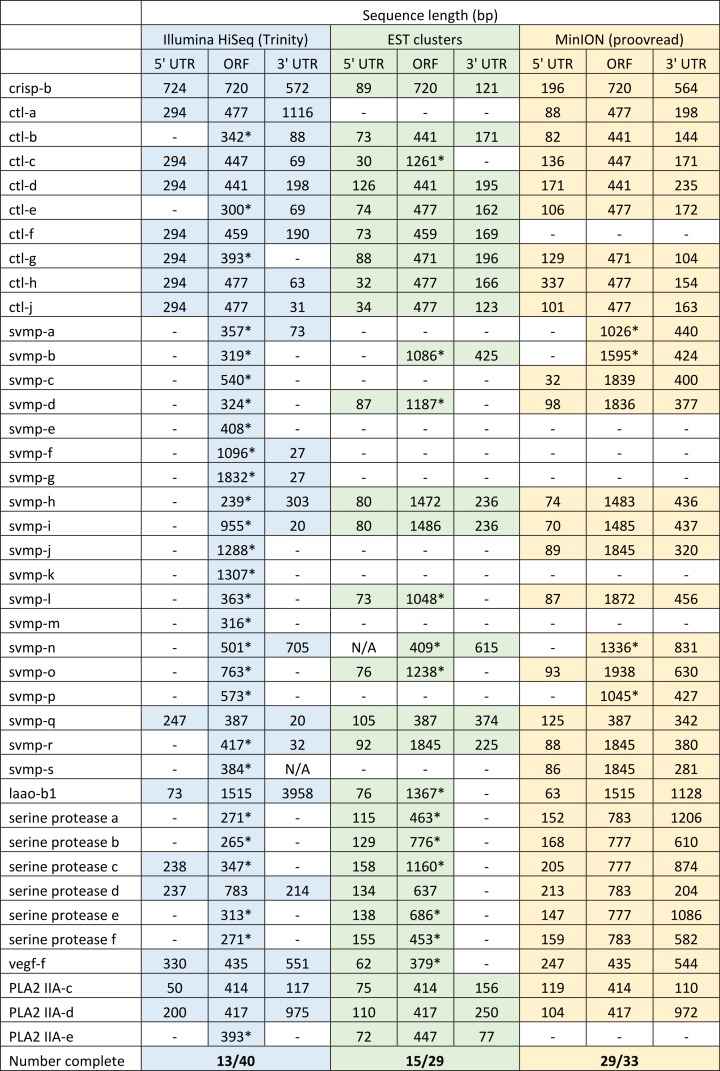
Comparisons of different sequencing approaches for the characterisation of transcripts encoding venom toxins in the painted saw-scaled viper (*Echis coloratus*) venom gland. The reference set of 40 candidate venom genes is derived from a Trinity (version trinityrnaseq_r2012-04-27) assembly of Illumina HiSeq data, where 13 transcripts contain the full open reading frame (ORF), although it is likely that the true number is lower, as the identical 5′ UTR length of *c-type lectin (ctl)* transcripts suggests misassembly. A set of EST clusters derived from 1,070 Sanger sequences from a pool of 10 individuals (Casewell et al., 2009) detects 29 of these transcripts, 15 of which contain the full ORF. Data generated using the Oxford Nanopore MinION, corrected using proovread, is able to detect 33 candidates, of which 29 contain the full ORF. Incomplete ORFs are indicated with an ^∗^.

**Figure 6:**
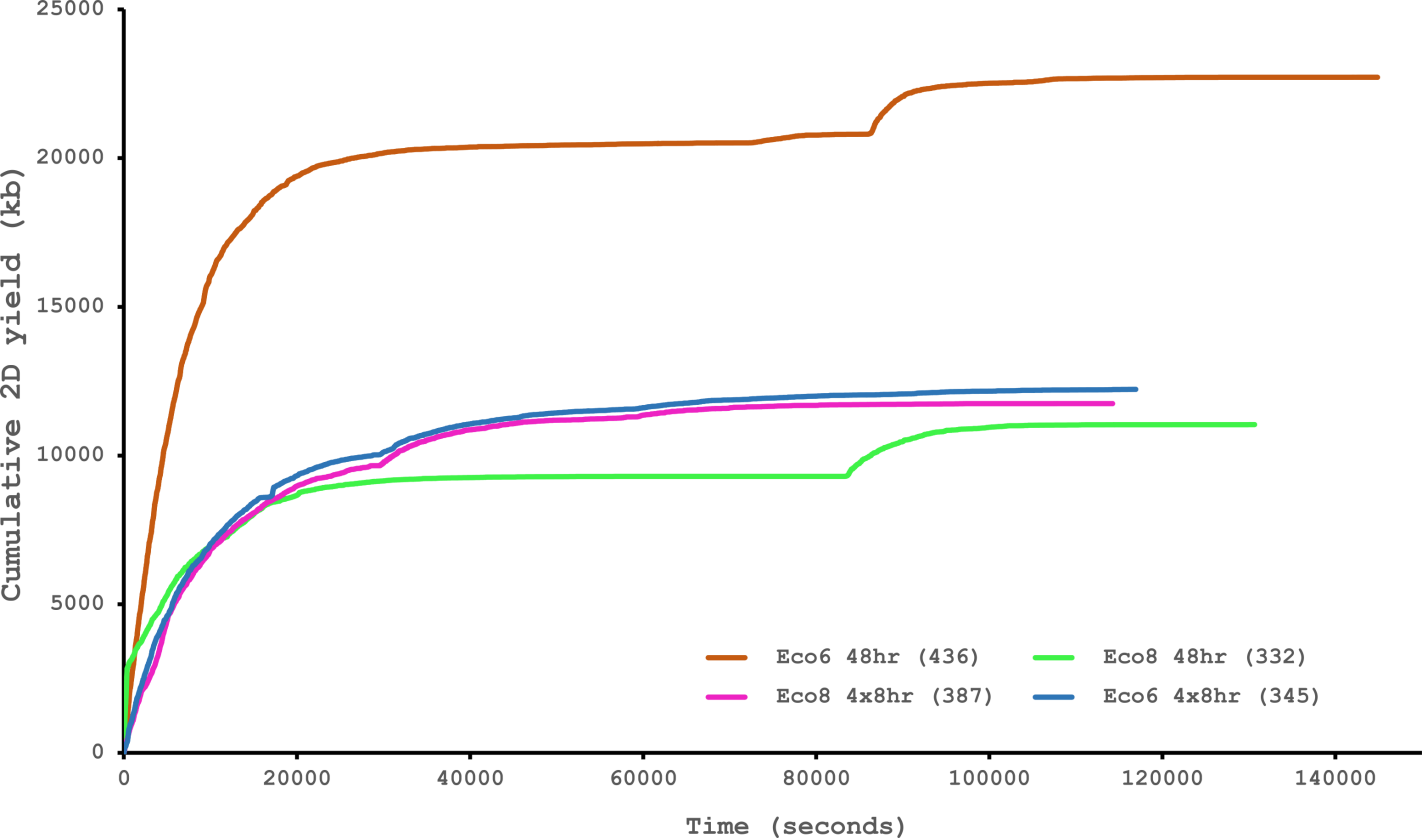
Data acquisition of 2D reads during four runs of the Oxford Nanopore MinION, using cDNA derived from two individuals (Eco6 and Eco8). Both the standard 48 h protocol (including a remux after 24 h) and a set of modified run scripts that perform four re-muxes at 8 h intervals were used. This latter approach yields a much smoother data acquisition profile. The number of pores available at initial QC for each flowcell is given in brackets in the legend.

We have previously suggested that the venom of *E. coloratus* comprises products from 34 different genes, in 8 gene families (Hargreaves et al., 2014b). However, in order to gain a better appreciation of the utility of the MinION for characterising venom gland transcriptomes, we have expanded our analyses beyond only these genes to other members of the same gene families which we previously ruled out as contributing to venom toxicity based on low expression levels and/or a wider tissue expression pattern (Fig. 5). Our Trinity (version trinityrnaseq_r2012-04-27) assembly of Illumina HiSeq data was able to reconstruct 13/40 full length sequences (which we define as a full open reading frame and at least some 5′ and 3′ untranslated region (UTR) sequence). This number is slightly misleading however, as seven of the *c-type lectin* (*ctl*) genes have identical 294 bp 5′ UTRs and have therefore likely been misassembled, probably as a result of very high similarity in the region encoding the signal peptide. The Sanger-based EST clusters reconstruct 15 of the 29 detected genes (Fig. 5). Interestingly, despite its reputation for producing chimeric transcripts (Archer et al., 2014), we find little evidence of this in our Trinity dataset and in fact encounter such issues only in the EST dataset, where *vegf-f*, *serine protease b* and *c* and *c-type lectin c* appear to be comprised of concatenated reads. The Illumina-corrected MinION reads for the Eco6 sample provided full coding sequences for 29 of 33 genes detected. Sequence identity between the corrected reads and the Trinity reference was typically 99–100% across the aligned region, and this was often higher than that of the EST clusters, where sequence quality deteriorated towards the ends. We were also able to identify putative splice variants using the MinION data that had not been recovered by either of the other two approaches. Although we did not detect all target transcripts, this was not unexpected for a variety of reasons. Firstly, the Illumina Trinity assembly reference dataset was assembled from several individuals at different time points during venom synthesis following milking and so certain genes may not be expressed in the samples used for our MinION experiments, and secondly, our analysis of the MinION dataset is based on only 40, 952 high-quality reads, whereas the Illumina data for the two samples comprised 52,179,724 paired-end reads (10,513,367,160 bp). Investigation of the effect of sequencing depth on the characterisation of snake venom gland transcriptomes using sub-assemblies of existing data (File S2) suggests that assemblies based on around 8 million 100 bp paired-end reads are able to return BLAST matches to all candidate genes. It is therefore truly exceptional that our much smaller amount of MinION data is able to provide not just matches, but full coding sequences for such a large number of venom genes in our study species. Although developed primarily to boost sequence production at the late stages of flow-cell use, we find that the modified 4 × 8 h run scripts produce a much smoother data acquisition profile (Fig. 6) and it seems likely that further refinements in this area will greatly improve data generation. The largest contributor to total sequence output however seems to be the number of available pores on each flowcell (Table 1 and Fig. 6) and greater consistency in this area, together with planned future increases to the number of pores per flowcell and the speed at which DNA traverses the pore will greatly increase the amount of data generated per flowcell. As an example of the speed at which the MinION and its associated technology and reagents are developing, we used the latest versions of Metrichor and 2D basecalling workflow (version 2.26.1 and 1.14 respectively) to re-analyse the Okinawa habu (*Protobothrops flavoviridis*) venom gland data that Mikheyev & Tin (2014) produced using an amplicon sequencing kit (most likely DEV-MAP001) and R6 flowcells. Despite less than a year separating our and their experiments, the runs that we performed using R7.3 flowcells using the 2D cDNA sequencing protocol with Nanopore Sequencing Kit SQK-MAP005 generated (roughly) 20–45 times as many reads; 50–100 Mb more total sequence; 450–1,000 times as much high-quality data and 500–1,000 times as much high-quality sequence. These figures clearly demonstrate the rapid pace of development of the Oxford Nanopore MinION to date.

## Conclusions

Until relatively recently it seemed as though DNA sequencing was coming to be dominated by a single company, and a single platform (or at the very least, a closely related family of platforms), with a particular focus on generating an ever-increasing number of human genome sequences. Indeed, the Illumina HiSeq X Ten system has been engineered to *only* be able to sequence human genomes and the required $10 million outlay restricts the number of potential purchasers significantly. Benchtop systems such as the Illumina MiSeq are more affordable and are becoming increasingly common at the research group or institutional level, although they still require a not-insignificant initial outlay and ongoing maintenance and update programs. Against this background, the Oxford Nanopore MinION has the potential to be a truly disruptive technology, offering long reads (in theory limitless, but in practise determined by the size of DNA fragments provided by the user), low and flexible pricing and portability. Planned or ongoing updates to the MinION, such as the release of the MinION MkI, new flowcells with increased numbers of pores, “fastmode” sequencing to increase output and automated sample preparation techniques will go some way to enabling the MinION to meet its full potential, but we predict that the greatest advances will come from improvements to the basecalling algorithms and the reduction of errors. However, hybrid approaches combining MinION data with shorter, more accurate Illumina reads are clearly already effective and can produce a fully circularised bacterial genome for around £500 (Risse et al., 2015), and *de novo* error-correction approaches have been shown to be possible in at least some cases (Loman, Quick & Simpson, 2015). For our purposes, a hybrid approach to error correction provided full coding sequences for a large number of venom toxin encoding genes, and was superior to both Illumina-only approaches and Sanger-based ESTs.

We estimate that the current full economic cost per Gb of sequence using the MinION is around £1,000, compared to around £40 per Gb for the Illumina HiSeq (and as low as £7 per Gb for the HiSeq X Ten). Generating even 1 Gb of sequence using 700–800 bp Sanger reads is far beyond the scope of most researchers. However, an EST approach, whilst inevitably low-throughput, does have the advantage of generating a physical, plasmid-based resource, where sequences (clones) of interest can be revisited, used in subsequent experiments (e.g., functional assays) and shared, all without the need for various rounds of primer design, PCR and cloning. In terms of both cost and output, and especially for experiments involving mapping reads to a high-quality, well-annotated reference genome, short-read (typically 150–300 bp) Illumina platforms currently have the edge, although for *de novo* assembly of highly-similar paralogous genes (such as those expressed in snake venom glands) they have their limitations. We therefore suggest that, in the absence of reference genomes, hybrid approaches based on smaller numbers of error-prone long reads and high numbers of highly-accurate short reads, will become the default method for the characterisation of transcriptomes from a wide range of species. The Pacific Biosciences RSII platform also offers long reads (at least 10 kb, and often longer), with a similar error-rate to the MinION, and so may also be useful for characterising full length transcripts from snake venom glands (certainly, the imminent introduction of the smaller, more affordable Pacific Biosciences Sequel is likely to make this platform a much more attractive proposition), although the size-selection steps in the current Iso-Seq protocol may lead to the loss of some transcripts.

Without doubt, recent (and planned) advances in technology, chemistry, error-correction and analysis across both short and long-read platforms will lead to a vast improvement in the quality of both genome and transcriptome sequences, and will open exciting new avenues of research for those of us that work on (more interesting) non-model species.

## Supplemental Information

10.7717/peerj.1441/supp-1 File S1 *De novo* error correction using Nanocorrect

10.7717/peerj.1441/supp-2 File S2 Subassemblies of venom gland Illumina HiSeq data

## Additional Information and Declarations

### DNA Deposition

The following information was supplied regarding the deposition of DNA sequences: Raw MinION venom gland data has been deposited in the European Nucleotide Archive under study number PRJEB10285 (Eco6 48 h run ERR985427; Eco6 4 × 8 h run ERR986484; Eco8 48 h run ERR985428; Eco8 4 × 8 h run ERR985429).
