# Supplementary material for: Assessing the utility of the Oxford Nanopore MinION for snake venom gland cDNA sequencing
Source: PeerJ. 2015 Nov 24;3:e1441. doi: 10.7717/peerj.1441 (PMC4662598; doi:10.7717/peerj.1441)
Supplement: File S1 [file peerj-03-1441-s001.pdf]

Supplementary file 1: *de novo* error correction using Nanocorrect

Supplementary Table S1. Correction of pooled Eco6 nanopore sequence data using up to five rounds of correction using Nanocorrect [1]. Little improvement is seen after the second round, although the number of sequences is much reduced as a result of this process.

	**Nanocorrect correction**				
	**Round 1**	**Round 2**	**Round 3**	**Round 4**	**Round 5**
Alignment length (bp)	9,948,507	5,807,383	4,245,548	3,681,607	3,363,671
Matches	9,623,049	5,660,507	4,140,907	3,591,236	3,280,638
Mismatches	98,888	40,682	31,202	26,821	25,011
Insertions	75,180	26,494	18,393	15,983	14,799
Deletions	226,570	106,194	73,439	63,550	58,022
Total Errors	400,638 **(4.0%)**	173,370 **(3.0%)**	123,034 **(2.9%)**	106,354 **(2.9%)**	97,832 **(2.9%)**
