# Supplementary material for: Assessing the utility of the Oxford Nanopore MinION for snake venom gland cDNA sequencing
Source: PeerJ. 2015 Nov 24;3:e1441. doi: 10.7717/peerj.1441 (PMC4662598; doi:10.7717/peerj.1441)
Supplement: File S2 [file peerj-03-1441-s002.pdf]

Supplementary file 2 - Sub-assemblies of the *Echis coloratus* venom gland transcriptome

In an attempt to determine the minimum required amount of sequencing to fully sequence and assemble the venom gland transcriptome of *Echis coloratus*, sub-sets of RNA-seq reads were extracted and assembled (Table S2). Paired venom gland reads were first interleaved using the `shuffleSequences.pl` perl script (part of the Velvet *de novo* assembly program [1]) so that each read pair was maintained during sub-sampling. Using the commands `head` and `tail`, 3 sub-sets (designated as "head", "middle" and "tail") of either 2, 4, 8 or 10 million reads were taken from an RNA-seq dataset containing 44,678,609 paired-end reads. These data were assembled using Trinity [2,3], with parameters set to run as a single-end read dataset (as there is only one .fastq input file), but with the added command-line parameter `--run_as_paired` to indicate that the data contains paired-end data.

Supplementary Table S2. Assembly metrics for sub-assemblies of the venom gland transcriptome of *Echis coloratus*.

Sub-sample	Sample size (million reads)	Total number of contigs	Number of contigs ≥300nt	Total length (nt)	Max. contig size (nt)	Contig N50 (nt)
HEAD	2	24,585	14,744	10,302,850	7,474	808
	4	34,990	22,184	17,605,771	7,860	1,023
	8	45,207	30,121	27,542,537	11,824	1,293
	10	48,349	32,660	31,623,176	11,824	1,420
MIDDLE	2	23,915	14,229	10,036,594	7,840	837
	4	34,383	21,736	17,282,856	8,970	1,027
	8	44,759	29,946	27,116,697	11,738	1,279
	10	47,832	32,451	30,985,872	11,752	1,387
TAIL	2	24,170	14,513	10,059,952	8,547	810
	4	34,735	21,994	17,315,514	8,165	1,004
	8	44,956	29,988	27,283,356	11,803	1,284
	10	48,116	32,535	31,022,314	11,805	1,382

Local blast surveys were then carried out using BLAST+ version 2.2.27 [4] to identify previously characterised putative toxin genes in *E. coloratus.* The majority of transcripts encoding putative toxin genes appear to be present in venom gland transcriptome assemblies generated from only 2 million paired-end reads (here presence is defined as the transcript being found in all three (Head/Middle/Tail) sub-assemblies) (Table S3).

Supplementary Table S3. Presence/absence of putative toxin transcripts in sub-assemblies of the venom gland transcriptome of *Echis coloratus.* Detected transcripts are shaded, transcripts not found are shaded grey. H, head; M, middle; T, tail.

	Sub-sample size and position											
	2 million reads			4 million reads			8 million reads			10 million reads		
Gene	H	M	T	H	M	T	H	M	T	H	M	T
3ftx-a	+	-	+	+	+	+	+	+	+	+	+	+
3ftx-b	+	+	+	+	+	+	+	+	+	+	+	+
ache - transcript 1	-	+	-	+	+	+	+	+	+	+	+	+
complement c3	+	+	+	+	+	+	+	+	+	+	+	+
crisp-b	+	+	+	+	+	+	+	+	+	+	+	+
crotamine-like	+	+	+	+	+	+	+	+	+	+	+	+
c-type lectins a-k	+	+	+	+	+	+	+	+	+	+	+	+
cystatin e/m	+	+	+	+	+	+	+	+	+	+	+	+
dpp 3	+	+	+	+	+	+	+	+	+	+	+	+
dpp 4	+	+	+	+	+	+	+	+	+	+	+	+
esp-e1	+	+	+	+	+	+	+	+	+	+	+	+
ficolin	+	+	+	+	+	+	+	+	+	+	+	+
kallikrein	+	+	+	+	+	+	+	+	+	+	+	+
kunitz 1	+	+	+	+	+	+	+	+	+	+	+	+
kunitz 2	+	+	+	+	+	+	+	+	+	+	+	+
laao-a	+	+	+	+	+	+	+	+	+	+	+	+
laao-b1	+	+	+	+	+	+	+	+	+	+	+	+
laao-b2	+	+	+	+	+	+	+	+	+	+	+	+
lipa-a	+	+	+	+	+	+	+	+	+	+	+	+
lipa-b	-	-	-	+	+	+	+	+	+	+	+	+
ngf	+	+	+	+	+	+	+	+	+	+	+	+
PLA_2 IIA-c	+	+	+	+	+	+	+	+	+	+	+	+
PLA_2 IIA-d	+	+	+	+	+	+	+	+	+	+	+	+
PLA_2 IIA-e	+	+	+	+	+	+	+	+	+	+	+	+
PLA_2 IIE	-	-	-	-	+	-	-	+	+	-	-	+
plb	+	+	+	+	+	+	+	+	+	+	+	+
renin	+	+	+	+	+	+	+	+	+	+	+	+
serine protease a-f	+	+	+	+	+	+	+	+	+	+	+	+
svmp-a	+	+	+	+	+	+	+	+	+	+	+	+
svmp-b	+	+	+	+	+	+	+	+	+	+	+	+
svmp-c	-	-	+	-	+	+	+	+	+	+	+	+
svmp-d	-	-	-	-	+	+	+	+	+	+	+	+
svmp-e	+	+	+	+	+	+	+	+	+	+	+	+
svmp-f	+	+	+	+	+	+	+	+	+	+	+	+
svmp-g	+	+	+	+	+	+	+	+	+	+	+	+
svmp-i	+	+	+	+	+	+	+	+	+	+	+	+
svmp-j	+	+	+	+	+	+	+	+	+	+	+	+
svmp-k	+	+	+	+	+	+	+	+	+	+	+	+
svmp-m	+	-	+	+	+	+	+	+	+	+	+	+
svmp-n	+	+	+	+	+	+	+	+	+	+	+	+
svmp-p	+	-	+	+	+	+	+	+	+	+	+	+
svmp-q	+	+	+	-	-	+	+	+	+	+	+	+
svmp-t	+	+	+	+	+	+	+	+	+	+	+	+
vegf-a	-	-	-	+	-	+	+	+	+	+	+	+
vegf-c	-	-	-	-	-	-	+	-	+	+	+	+
vegf-f	+	+	+	+	+	+	+	+	+	+	+	+
waprin	+	-	+	+	+	+	+	+	+	+	+	+

As the number of reads used for assembly increases the mean length of the amino acid sequence encoded by the assembled transcript also increases, although there is only a 36 amino acid increase between 2 million and 10 million reads (Figure S1).

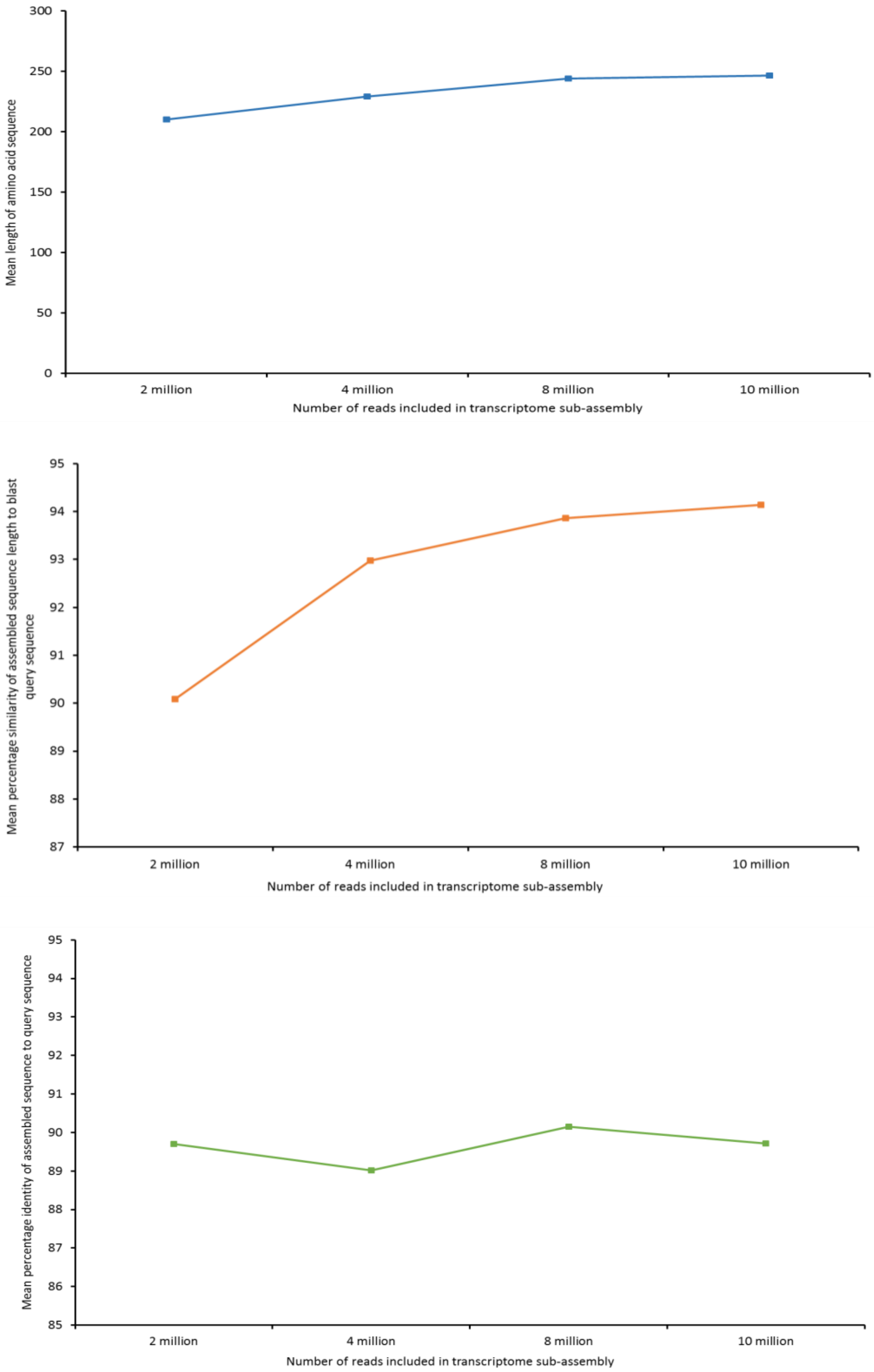

Supplementary figure S1. Analysis of sequence assembly quality based on local blast surveys using previously characterised amino acid sequences from *Echis coloratus* venom gland. Top - mean length of amino acid sequence matches in sub-assemblies, Middle - mean percentage length of query sequence covered by assembled sequence. Bottom - mean percentage similarity of assembled sequence to query sequence in sub-assemblies.

However, the number of contigs ≥300bp roughly doubles (Table S1), meaning considerably fewer contigs which are likely to be unplaced paired reads are present in the transcriptome assembly. To gain insight into how this increase in length relates to the quality of the assembled toxin transcript sequences, the percentage of the query sequence covered by the newly assembled sequence was calculated. Again there is only a minor improvement of 4% following an increase from 2 million reads to 10 million (Figure S1). The mean percentage similarity between assembled sequence and query sequence appears to be more variable across the sub-assemblies, with no apparent consistent improvement as the number of reads increases (Figure S1). As the query sequences used for local BLAST searches were obtained from an assembly of multiple *E. coloratus* venom gland datasets in order to represent an overabundance of sequencing, and the sub-assemblies were assembled from a different set of venom gland reads, it should be expected that not all blast alignments will have a 100% match between query and subject due to variation between individuals. However, a lower % identity would indicate that either sequencing errors were incorporated into the assembly or there has been a misassembly, both likely due to a reduced depth of sequencing coverage.

Conclusion

Around 8 million reads appears to be sufficient sequencing depth to capture all putative toxin-encoding transcripts to a suitable assembly quality. The Illumina HiSeq2500 sequencing platform can currently produce 300-400 million 100nt paired-end reads in “high output” mode, or 200-300 million 150nt paired-end reads in “rapid run” mode. With this in mind, and 8 million paired-end reads assumed to be the minimum sequencing depth required to fully capture all putative toxin transcripts, it is possible to sequence ~40 venom gland libraries on one sequencing lane of the Illumina HiSeq2500 (in “high output” mode).

Supplementary references

1. Zerbino DR, Birney E. 2008. Velvet: Algorithms for de novo short read assembly using de bruijn graphs. *Genome Res* **18:** 821-829.

2. Grabherr MG, Haas BJ, Yassour M, Levin JZ, Thompson DA, Amit I, Adiconis X, Fan L, Raychowdhury R, Zeng Q. 2011. Full-length transcriptome assembly from RNA-seq data without a reference genome. *Nat Biotechnol* **29:** 644-652.

3. Haas BJ, Papanicolaou A, Yassour M, Grabherr M, Blood PD, Bowden J, Couger MB, Eccles D, Li B, Lieber M. 2013. De novo transcript sequence reconstruction from RNA-seq using the trinity platform for reference generation and analysis. *Nat Protocols* **8:** 1494-1512.

4. Camacho C, Coulouris G, Avagyan V, Ma N, Papadopoulos J, Bealer K, Madden TL. 2009. BLAST+: Architecture and applications. *BMC Bioinformatics* **10:** 421.
